# Dietary and Supplementation Strategies for Modulating Gut Microbiota: A Narrative Review

**DOI:** 10.3390/foods15142561

**Published:** 2026-07-21

**Authors:** Natalia Ekstedt-Biskot, Dominika Jamioł-Milc, Klaudia Melkis, Joanna Pieczyńska

**Affiliations:** 1 Department of Biochemical Science, Pomeranian Medical University in Szczecin, Broniewskiego 24, 71-460 Szczecin, Poland; natalia.ekstedt@gmail.com; 2 Department of Human Nutrition and Metabolomics, Pomeranian Medical University in Szczecin, Broniewskiego 24, 71-460 Szczecin, Poland; klaudia.melkis@pum.edu.pl; 3 Department of Food Science and Dietetics, Wroclaw Medical University, Borowska 211, 50-556 Wrocław, Poland; joanna.pieczynska@umw.edu.pl

**Keywords:** microbiota, probiotics, prebiotics, dietary therapy, disease prevention

## Abstract

The composition and balance of the gut microbiota are important for human health, as they are involved in metabolic processes, immune regulation, intestinal barrier function, and gut–brain communication. This narrative review summarizes current evidence on dietary and supplementation-based strategies for modulating the gut microbiota, with particular attention to dietary fiber, fermented foods, prebiotics, probiotics, synbiotics, and postbiotics. The review discusses their potential mechanisms of action, including effects on microbial composition, short-chain fatty acid production, intestinal barrier integrity, immune responses, and metabolic homeostasis. Particular emphasis is placed on the context-dependent nature of microbiota modulation, as the effects of dietary and supplementation strategies may vary according to baseline microbiota composition, health status, habitual diet, probiotic strain, dose, and intervention duration. Overall, current evidence suggests that microbiota-targeted nutritional strategies may support gut homeostasis and selected health-related outcomes; however, their effects are not universal and require more personalized and better-controlled approaches in future research.

## 1. Introduction

Currently, the term “microbiota” means “the collection of all microorganisms living in a specific environment (human body–host), namely bacteria, fungi, viruses and archaeons, while the term “microbiome” means the collection of their genomes” [1].

It is noteworthy that the number of bacterial cells in the human body is currently considered to be of the same order of magnitude as the number of human cells. Revised estimates suggest approximately 3.8 × 10^13^ bacterial cells and 3.0 × 10^13^ human cells in a 70 kg reference adult male, corresponding to a bacteria-to-human cell ratio close to 1:1. The total bacterial mass has been estimated at approximately 0.2 kg, which is substantially lower than earlier, frequently repeated estimates. The gut microbiota may exert beneficial effects on the host when its composition and metabolic activity remain balanced and when potentially pathogenic microorganisms do not predominate [2].

The action of microbiotic bacteria is extensive, but supporting digestive processes and gaining energy from digested food is their main task [1,2,3]. Food that has not been digested is broken down by fermentation carried out by bacteria in the large intestine. The main bacteria inhabiting the digestive tract are *Firmicutes* and *Bacteroidetes*. Aerobic bacteria, such as *Enterococcus* and *Enterobacteriaceae*, are in a distinct minority [2].

The composition of the intestinal microbiota is different for each person. The microbiota of the human body forms during the first hours after birth and then undergoes constant changes during life, affecting immunity and overall health [4].

It has also been suggested that it may have a role in the development of metabolic diseases [4]. Indeed, it has been noted that some disease entities are associated with abnormal composition of the gut microbiota and also imbalances in the brain–gut axis. Such diseases include inflammatory bowel diseases, neurodegenerative diseases such as Parkinson’s and Alzheimer’s, cardiovascular diseases, allergies, obesity and diabetes [5]. In the case of neurodegenerative diseases, including Parkinson’s disease and Alzheimer’s disease, disease-specific alterations in gut microbiota composition have been reported, although the direction and biological meaning of these changes may differ depending on the disease, population, and taxa analyzed [6].

Chronic heart failure is characterized by an increase in *Escherichia coli*, *Klebsiella pneumonia*, and *Streptococcus viridans*. Patients with established atherosclerosis have been reported to show an increased abundance of *Lactobacillus* and a decreased abundance of *Roseburia*. In hypertension, an increased *Firmicutes*-to-*Bacteroidetes* ratio has been observed, while type 2 diabetes and obesity have also been associated with an increased abundance of *Firmicutes* and a decreased abundance of *Bacteroidetes* [7].

Over the past few years, it has been shown that communication between the gut microbiota and the central nervous system (CNS) is via the brain–gut axis. Information is transmitted to the CNS by gut microbial metabolites such as short-chain fatty acids (SCFAs), secondary bile acids and tryptophan metabolites. The CNS, in turn, can interact with the gut microbiota through catecholamines and also endocrine mediators (e.g., cytokines). The vagus nerve and spinal cord are also important communicators. This axis has a huge impact on the production of microglia in the brain, support of neuronal signaling, efficient functioning of the blood–brain barrier, changes in gut physiology and also the composition and activity of gut bacteria. An imbalance in the brain–gut axis is often associated with the occurrence of a wide variety of homeostatic disorders in the human body [8].

The intestinal microbiota coordinates the state of homeostasis in the gut by influencing epithelial regulation, the mucosal immune system, and the neuromuscular activity of the gut. In addition, its adequate colonization by probiotic bacteria reduces the concentration of pro-inflammatory cytokines in the body, thereby reducing the permeability of the intestinal barrier and the inflammation occurring in the host [9,10,11].

When there is a quantitative and qualitative imbalance in the composition of the microbiota, dysbiosis occurs. This is a condition in which both the quantity and quality of organisms residing in the intestine are adversely modified. In addition to differences in the composition of the microbiota, changes in the function of certain strains of bacteria are also observed. Although intestinal dysbiosis can occur at any stage of life, recent studies indicate a link between its early onset and the later development of diseases. This underscores the need for a more in-depth understanding of the mechanisms leading to its formation. A key element of prevention efforts against the growing number of NCDs is a better understanding of the interplay between host genetics, gastrointestinal maturation and environmental factors. The gut microbiome shows the greatest plasticity during the first years of life, making it a period of particular vulnerability to microbial imbalances. Risk factors include the type of diet and antibiotic therapies used, but also environmental and genetic factors that increase the host’s predisposition to intestinal microbiota disorders. It is assumed that dysbiosis can occur through a number of risk factors occurring over an extended period of time [12]. The state of dysbiosis is so unfavorable to the body that it can contribute to or accompany many diseases, such as diabetes, obesity, asthma, some allergies, and inflammatory bowel diseases (IBD) [3,5,6,7,8]. Additionally, the role of gut dysbiosis as a potential factor in the onset of neurodegenerative diseases is increasingly being recognized. Current studies indicate that patients with Parkinson’s disease exhibit an altered relative abundance of bacterial species in the gut, either increased or decreased [13]. In individuals with Parkinson’s disease, the gut microbiota shows an intensified degradation of proteins, leading to an increased production of harmful metabolites such as phenylacetylglutamine and p-cresol. Elevated levels of these compounds, along with alterations in gut bacterial composition, are strongly associated with constipation, a common symptom in affected patients [14]. Meta-analyses have identified common features defining dysbiosis in Parkinson’s disease, including an increased relative abundance of genera *Akkermansia*, *Lactobacillus*, *Catabacter*, and *Bifidobacterium*, as well as families *Bifidobacteriaceae*, *Akkermansiaceae*, *Ruminococcaceae*, *Verrucomicrobiaceae*, and *Christensenellaceae*. Conversely, a decreased abundance of genera *Faecalibacterium* and *Roseburia*, along with families *Prevotellaceae* and *Lachnospiraceae*, has been observed [15,16,17]. Although *Lactobacillus* and *Bifidobacterium* are commonly regarded as beneficial or probiotic-associated taxa, their increased relative abundance in Parkinson’s disease should be interpreted in a disease-specific ecological context. In this setting, their enrichment has been reported together with the depletion of major SCFA-producing bacteria, such as *Faecalibacterium* and *Roseburia*, suggesting that these alterations may reflect broader dysbiosis, altered gut transit, constipation-related changes, intestinal inflammation, or compensatory microbial changes rather than a uniformly protective profile [14,15,16,17]. A meta-analysis aimed at identifying differences in gut microbiota composition between Alzheimer’s disease patients and healthy individuals revealed a noticeable decline in bacterial species diversity. Notably, the genus *Bacteroides* was often found in increased abundance, particularly in the American cohort. In contrast, individuals with mild cognitive impairment exhibited a significant increase in bacteria from the genus *Phascolarctobacterium* [18].

Despite extensive research on dietary strategies, prebiotics, probiotics, synbiotics, and postbiotics, the available evidence remains heterogeneous and depends on the population studied, baseline microbiota composition, health status, intervention duration, dose, and the specific microbial strains or substrates used. Therefore, this narrative review aims to summarize and organize current evidence on dietary and supplementation-based approaches to gut microbiota modulation, with particular attention to their mechanisms of action, clinical relevance, and limitations. Rather than presenting these strategies as universally effective, the review emphasizes their context-dependent effects and the need for more personalized approaches in future nutritional research and practice.

The aim of this narrative review was to summarize current evidence on the interactions between dietary factors, prebiotics, probiotics, synbiotics, postbiotics, and the gut microbiota, and to discuss their potential relevance in selected health conditions.

## 2. Probiotics

According to the World Health Organization (WHO) and Food and Agriculture Organization of the United Nations (FAO) definitions, a probiotic is defined as “a live microorganism administered in adequate amounts that provides health benefits to the host.” In recent years, however, researchers have noted that not only live bacteria, but also their metabolites and dead/inactivated bacterial cells can significantly affect the nutritional status of the intestinal microbiota and also processes in the human body [19]. Therefore, in 2019, the International Scientific Association for Probiotics and Prebiotics (ISAPP) introduced the definition of postbiotic as a “preparation of inanimate microorganisms and/or their components that confers a health benefit on the host”. Therefore, according to this definition, effective postbiotics must contain inactivated microbial cells or cell components, with or without metabolites [20].

**Figure 1:**
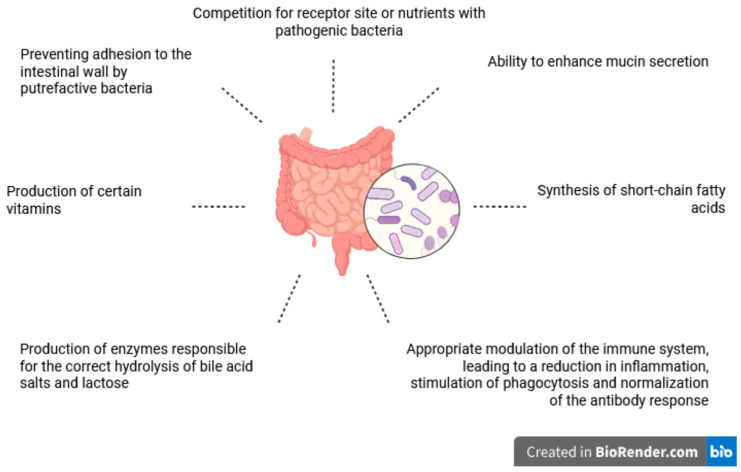
Activity of commensal bacteria in the human gut [2,21]. Created in https://BioRender.com.

Depending on the strain or group of strains, probiotics show different effects on the body, but their common feature is colonization of the large intestine, leading to the maintenance of homeostasis inside the body. Probiotic bacteria are designed to change the reaction of the colon environment to an acidic one. Such a reaction of the environment is beneficial through the reduced possibility of pathogenic bacteria multiplying in it, while probiotic bacteria populate the intestine [2,3]. Probiotic bacteria can restructure the human bacterial microbiota. The mechanisms by which this process can occur are shown in Figure 1.

A mechanism that requires special explanation is the synthesis of short-chain fatty acids (SCFAs). This is because they are important for intestinal function and condition [22]. Short-chain fatty acids, which are the final metabolites of fiber metabolism by bacteria residing in the intestine, include acetic acid, butyric acid, propionic acid, valeric acid, and caproic acid. Their concentration in the intestine depends on several factors, such as current health status, physical activity, or diet [22,23]. The most significant variable affecting the concentration of SCFAs produced in the intestinal lumen is the amount of dietary fiber consumed. Nevertheless, regardless of the level of supply of substrates necessary for SCFA production, the amount of SCFAs is lower in the intestinal lumen of individuals struggling with certain disease entities compared with a healthy person [24].

The production of individual SCFAs is associated with different groups of intestinal bacteria. Acetate is produced by a broad range of gut microorganisms, including *Bifidobacterium*, *Bacteroides*, and *Akkermansia*. Propionate production is commonly associated with *Bacteroides* and selected members of the *Lachnospiraceae* family, depending on the metabolic pathway involved. In contrast, butyrate is mainly produced by bacteria from the Firmicutes phylum, especially *Faecalibacterium*, *Roseburia*, *Eubacterium*, *Coprococcus*, and other members of the *Lachnospiraceae* and *Ruminococcaceae* families. However, SCFA production depends not only on the presence of specific taxa, but also on substrate availability, dietary fiber intake, and cross-feeding interactions within the gut ecosystem [25,26,27].

Short-chain fatty acids positively affect the integrity of the intestinal barrier. However, the main role is played by butyric acid, which is a source of energy for colonocytes—epithelial cells of the intestine. It also inhibits kappa factor B (factor kappa B, NF-kappaB) and histone deacetylase, which translates into its anticancer and epigenetic effects. SCFAs provide up to 60% of the energy required by intestinal lining cells. The extraintestinal actions of SCFAs relate to the modulation of the gut–brain axis through the ability of short-chain fatty acids to cross the blood–brain barrier and playing an important role in the maturation of immune cells in the bone marrow. Short-chain fatty acids can also exert tissue-specific effects by increasing the feeling of satiety, affecting the regulation of systemic immunity, and increasing insulin sensitivity [23,24,25].

Different SCFAs have a variety of functions, which is why it is important to maintain adequate levels of each in the body. Acetic acid, which is a crucial substrate for cholesterol synthesis, along with propionate, plays a significant role in the regulation of lipid metabolism [26]. Propionic acid, on the other hand, is an essential component of gluconeogenesis occurring in the liver [27]. Butyric acid exhibits anti-inflammatory effects, provides a source of energy for enterocytes and promotes intestinal regeneration [28]. Valeric acid, as a histone deacetylase inhibitor, may exhibit anti-tumor, anti-inflammatory, anti-diabetic and immunomodulatory properties [29]. Caproic acid, in contrast, promotes the methylation process and improves the absorption of selected components in the intestine [30].

The effects of SCFAs on the body have been widely reported in the literature. They have been shown to interact with G-protein-coupled receptors and are involved in inhibiting histone deacetylation, which translates into the regulation of many biological processes. Thus, they contribute to enhancing regulatory T lymphocyte responses and increasing the body’s immune tolerance, while showing anti-inflammatory and antimicrobial effects. In addition, they stimulate mucus secretion, help maintain the integrity of the intestinal barrier, and promote the synthesis of dendritic cell precursors in the bone marrow [31,32]. All of these processes play a key role in the formation of the intestinal barrier in children. The results of a study by Homann et al. in 2023 [33] indicate that higher concentrations of SCFAs in the intestine, especially butyrate, may reduce the risk of *Salmonella* infection in test animals [33].

An interesting phenomenon is the so-called butyrate paradox, which is that when the body is healthy, colonocytes feed mainly on butyric acid, but in the case of cancer, their energy source becomes glucose. As a result, butyric acid is not consumed by them but is instead stored to more effectively inhibit histone deacetylase in the diseased tissues [34].

The dosage of probiotic preparations has not yet been precisely determined. However, some probiotic strains have a recommended dose and duration of therapy. Therefore, no single generally recommended probiotic dose can be proposed for all strains, products, populations, and clinical indications. Probiotic efficacy is strain-specific and depends on several factors, including formulation, viability at the end of shelf life, route of administration, target population, health condition, and duration of intervention. In clinical studies, probiotic doses are commonly used within the approximate range of 10^8^–10^11^ CFU/day; however, this range should be interpreted as a commonly studied range rather than a universal recommendation. Thus, probiotic dosage should always be considered in relation to the specific strain, product, and expected clinical outcome [35,36,37,38,39,40,41,42,43,44,45].

Information on the results of studies on the effectiveness of doses and duration of probiotic intervention in various diseases is presented in Table 1. The use of an appropriate type of preparation (single or multi-strain probiotics) in patients with acute gastroenteritis or type 2 diabetes mellitus or type 2 diabetes mellitus with diabetic nephropathy after only 1 week of intervention (maximum intervention time 12 weeks) resulted in a significant change in clinical parameters such as reduction in leukocyte concentration, reduction in inflammation, improvement of frequency of bowel movement, enhanced renal function and enhanced glycaemic control [35,36,37,38,39,40,41,42,43,44,45].

Additionally, meta-analyses examining the efficacy of probiotic supplementation indicate that, depending on the strain administered, they may carry a different therapeutic effect and be effective in different disease entities (Table 2). The use of both multi-strain probiotics and single-strain probiotics resulted in a reduction in body mass index, fasting blood glucose concentration, fasting insulin concentration, triglycerides concentration, Low-Density Lipoprotein concentration, and systolic blood pressure in patients with Type 2 Diabetes [47]. A recent systematic review and meta-analysis demonstrated modest but significant reductions in office systolic and diastolic blood pressure following long-term probiotic supplementation [48].

Selecting the correct probiotic therapy requires a detailed interview with the patient, as many different factors determine what form the product should take (multi-strain probiotics or single-strain probiotics). Criteria taken into account when selecting a probiotic include the patient’s existing or past illnesses, the type of treatment used, as well as the availability and efficacy of particular strains of probiotic bacteria demonstrated in studies [49].

## 3. Prebiotics

According to the International Scientific Association for Probiotics and Prebiotics (ISAPP) definition, “A dietary prebiotic is a selectively fermented ingredient that causes specific changes in the composition and/or activity of the gastrointestinal microbiota, thereby providing health benefits to the host.” The group of prebiotics includes fiber, inulin, resistant starch and also fructooligosaccharides (FOS) and galactooligosaccharides (GOS) [10]. Prebiotics can also be defined as the undigested parts of food beneficially affecting the host body by selectively stimulating the growth of certain bacteria residing in the colon. This results in the correct proliferation of the intestinal microbiota. Prebiotics also increase the metabolic activity of intestinal bacteria, thus leading to increased production of SCFAs [8].

Thecriteria that a compound must meet to be defined as a prebiotic [50]:

- Resistant to the acidic environment of the stomach;
- Not hydrolyzed by the host’s digestive enzymes;
- Not absorbed in the gastrointestinal tract;
- Fermented by intestinal bacteria;
- Can selectively stimulate the growth and activity of host intestinal bacteria [50].

### 3.1. Prebiotics of Natural Origin

Prebiotics of natural origin are those that are supplied with food. The first prebiotic provided to the body after birth is the oligosaccharides in breast milk. Thanks to them, the initial growth and proliferation of the newborn’s microbiota take place [51]. The group of compounds classified as prebiotics of natural origin is extensive. They include mixtures of low-molecular-weight oligosaccharides that do not undergo digestion in the gastrointestinal tract. Their ability not to be hydrolyzed by digestive enzymes in the upper digestive tract is due to the presence of glycosidic bonds. As a result, they reach the large intestine unchanged and can become a substrate for fermentation carried out by intestinal bacteria. Table 3 shows the characteristics of the various prebiotic compounds along with examples of their sources [50,51,52,53,54,55,56,57,58,59,60,61,62].

Prebiotics are also available on the market as dietary supplements, in which the active ingredient may be:

- Lactulose;
- Inulin;
- Fructooligosaccharides;
- Inulin and fructooligosaccharides;
- Dietary fiber;
- Resveratrol;
- Galacto-oligosaccharides and xylo-oligosaccharides;
- Arabinogalactans.

FOS supplementation of adult mice in a study conducted by Watanabe et al. [55] resulted in an increased abundance of *Bifidobacterium* bacteria which, in turn, was associated with a reduction in the severity of skin allergies. Similar conclusions were reached by Kivit et al. [56], who found that following the administration of a synbiotic consisting of short-chain GOS/long-chain FOS and *Bifidobacterium* breve M-16V was associated with increased galectin-9 levels in mice and also humans. Galectin-9 reduces mast cell degranulation and promotes Th1 and Treg responses. Therefore, consumption of the synbiotic was associated with a significant reduction in the occurrence of acute cutaneous allergic reactions [55].

Interestingly, it has been suggested that in addition to selectively stimulating microbiota to grow, prebiotics may also have direct anti-inflammatory effects on the body. In a study led by Zenhom et al. [57], it was shown that prebiotic intake was associated with reduced secretion of interleukin 12 (IL-12) in Caco-2 cells and also reduced expression of p35 protein, interleukin 8 (IL-8) and tumor necrosis factor α (TNF-α) [57].

Prebiotics may also play an important role in reducing the risk of Alzheimer’s disease. In the Hispanic population studied (Hispanics), fructans played a role. Increasing dietary fructan intake by as little as one gram was associated with a 24% reduction in AD risk (HR = 0.76, 95% CI 0.60–0.97; *p* = 0.03) [58].

### 3.2. Prebiotics and Synbiotics in Pharmaceutical Preparations

When prebiotics taken with food are insufficient, it may be necessary to take pharmaceutical preparations with prebiotic properties. The most common oral preparation is lactulose and inulin, which comes in the form of capsules, powder or herbs (i.e., traveler’s chicory, oman root or dandelion root) for brewing [59].

The results of a single-arm study with 26 healthy Japanese women involving a pre-intervention period and three two-week periods of lactulose supplementation (at 1, 2 and 3 g/day) with a two-week break between each show that even 1 g of lactulose per day exerts a prebiotic effect. It was also observed that the beneficial effect of lactulose on the gut microbiota was dose/day dependent. A two-week intervention of 1 g/day of lactulose caused an increase in fecal *Bifidobacteria* from 9.93 (±0.57) log CFU/g feces to 10.10 (±0.40) log CFU/g feces, a dose of 2 g/day of lactulose induced a change from 9.95 (±0.63) log CFU/g feces to 10.23 (±0.53) log CFU/g feces, while at 3 g/dz of lactulose from 10.09 (±0.51) log CFU/g feces to 10.38 (±0.28) log CFU/g feces [60]. The same team of Japanese researchers conducted a randomized, double-blind, placebo-controlled crossover study involving 52 healthy Japanese women to evaluate the prebiotic effects of lactulose supplemented at 2 g/day. The two-week intervention resulted in an increased number of *Bifidobacterium* (log CFU/g stool) in the stools of the women studied (9.53 ± 0.06) compared with placebo (9.16 ± 0.06) (Δ0.37 [95% CI 0.23–0.49], *p* < 0.0001) and significantly increased the percentage of *Bifidobacterium* (25.3 ± 1.4) compared with placebo (18.2 ± 1.4) (Δ7.1 [95% CI 2.9–11.4], *p* = 0.0014). Defecation frequency, number of days of defecation (days/week), stool consistency measured by the Bristol scale, stool volume and effort during defecation were also significantly improved, while no increase in flatulence was observed [61]. The cited studies suggest that supplementation with low doses of lactulose may have a positive effect on the composition of the intestinal microbiota, thus contributing to the regulation of defecation.

In contrast, the efficacy of inulin in modulating the composition of the intestinal microbiota was confirmed in a systematic review by Le Bastard et al. 2020 [62]. An analysis of the results of nine randomized or controlled human studies available to date found that supplementation with inulin isolated from chicory root, agave, or extracted from artichoke, at a dose of 5 to 20 g per day for a period of 7 days to 3 weeks, results in an increase in the abundance of *Bifidobacterium*, *Faecalibacterium*, *Anaerostipes* and *Lactobacillus*, and a decrease in the relative abundance of *Bacteroides*.

Synbiotic preparations, on the other hand, consist of probiotics and prebiotics, which should act synergistically with each other. Therefore, the composition of a synbiotic should be carefully considered so that the prebiotic that is selective for most bacteria acts favorably to the probiotic used in the formulation. The idea behind the synbiotic was to increase the effectiveness of applied probiotics entering the human gastrointestinal tract by increasing their chances of survival in the upper gastrointestinal tract and facilitating colonization within the colon, resulting in measurable health effects for the host [63].

The results of a 2024 meta-analysis of 16 studies show that synbiotics modulate the intestinal microbiota by increasing the number of *Lactobacilli* (SMD = 0.74; 95% Cl: 0.15, 1.33, *p* = 0.01; *I*^2^ = 69.4, *p* = 0.02), relative concentration (%) of *Bifidobacterium* (WMD = 0.97; 95% CI: 0.42, 2.52; *p* = 0.10; *I*^2^ = 93.0, *p* = < 0.001), *Bifidobacterium* count (SMD = 0.82, 95% CI: 0.13, 1.88, *p* = 0.06; *I*^2^ = 93.1, *p* < 0.001) and propionate concentration (SMD = 0.22; 95% CI: 0.02, 0.43, *p* = 0.03; *I*^2^ = 0.0, *p* = 0.79) compared with the control group [64].

## 4. Dietary Therapy to Promote Adequate Growth and Composition of the Intestinal Microbiota

**Figure 2:**
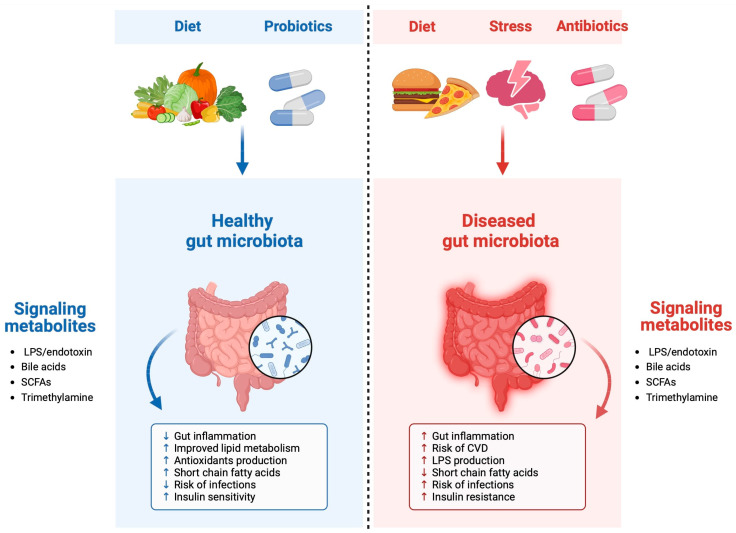
Influence of diet and lifestyle on gut microbiota [65,66,70]. ↑: increase; ↓: decrease; Created in https://BioRender.com.

Dietary features that adversely affect the state of the intestinal microbiota are a diet plentiful with red meat, simple carbohydrates, high amounts of saturated fatty acids, and low fiber intake. The above-mentioned products are also the main components of a Western-type diet, which studies have shown to have a strong pro-inflammatory effect on the body, while adversely affecting the state of the human microbiota [65,66,67,68,69]. During its use, the composition of microorganisms and their metabolic activity changes. It is supposed that these changes may be linked to the increasing incidence of chronic diseases, a significant increase of which has been observed in highly developed countries, where a Western-type diet is followed by a significant part of the population [66,70]. The changes occurring in the gut microbiota under the influence of diet are shown in Figure 2.

In order to see how, and over what period of time, changes in the composition and activity of microorganisms residing in the human large intestine can occur, a study was conducted in which respondents were divided into two groups [70]. One group consumed a plant-based diet rich in legumes, cereals, vegetables and fruits. The other group was introduced to a dietary model in which animal products such as meat, eggs and cheese predominated. There were no other restrictions on consumption beyond the main group of products. The study volunteers, aged 21 to 33, were told to consume the designated diet for five days. Participants were surveyed for four days before, during and six days after the study to closely analyze changes in their intestinal microbiota. It is worth noting that the daily diet of each subject differed significantly from the one introduced for the duration of the experiment. The amount of fiber consumed in the group on an animal diet was almost zero, while in the group on a plant-based diet it reached 25 g per 1000 kcal. After quantitative analysis of the microorganisms present in the intestine of each patient from all days of the study, it was shown that the greatest differences from the baseline microbiota occurred in the group on the animal diet. Interestingly, these could be observed just one day after the researchers noted that the food content from the time of the study was in the large intestine. This was possible thanks to special dyes that make it easier to track the components of meals in the body. The return to the pre-test microbiota occurred two days after switching back to the original diet. By analyzing the fecal SCFA and bacterial content, the researchers observed that the type of diet consumed affects the metabolic activity of the microorganisms. In the group of people on a plant-based diet, carbohydrate fermentation predominated, while the group consuming animal products was dominated by protein fermentation products. In those on the animal diet, bacteria with the ability to grow in higher concentrations of bile definitely predominated due to the increased amount of fat consumed. The concentration of the secondary bile acid deoxycholic acid (DCA), which is the final metabolite of the microorganisms residing in this group of subjects, also increased. The concentration of DCA is positively correlated with the incidence of liver cancer and with inhibition of the growth of *Bacteroides* and *Firmicutes*. An increase in the concentration of bile salt acids in the feces of people on an animal diet correlated with a significant increase in the abundance of *Bilophila wadsworthia*, a bacterium that reduces sulfate to hydrogen sulfide, thus causing increased inflammation in the intestine. This suggests that *Bilophila wadsworthia* may contribute to inflammatory bowel disease and other diseases involving chronic inflammation and impaired immune barriers in the gut. The entire study was designed to demonstrate how the activity and composition of the intestinal microbiota change depending on diet [70].

Previous evidence and reviews suggest that a Western-type diet, rich in animal protein and fat and low in dietary fiber, may disrupt the intestinal microbiota by reducing beneficial commensal bacteria, including *Bifidobacterium* and *Eubacterium* [67,68]. Western dietary patterns and processed animal-derived foods may also be associated with increased exposure to N-nitrosamines, which have been evaluated by EFSA as food-related risk factors [71].

The results of a 2023 randomized clinical trial conducted by Corbin et al. [69] show significant differences between the composition of the gut microbiome of people following a Western diet (WD) and those consuming minimally processed foods, high in dietary fiber and resistant starch. Individuals on the Microbiome Enhancer Diet (MBD) had higher relative abundance of dietary fiber-degrading bacteria, i.e., *Prevotella copri*, uncharacterized *Prevotella* and *Lachnospira pectinoschiza* (Q = 1.46 × 10^−6^, 0.0005 and 0.001, respectively) or butyrate-producing bacteria, i.e., *Lachnospira pectinoschiza*, *Eubacterium eligans* and uncharacterized *Oscillibacter* (CAG_241 and 57_20) (Q = 1.46 × 10^−6^, 0.001, 7.44 × 10^−7^, 0.01 and 2.27 × 10^−7^). On the other hand, in subjects eating according to the Western dietary model, higher relative abundance of bacteria obtaining energy from the fermentation of host glycans, simple sugars or fermentation products (mainly CO_2_ and H_2_) produced by other gut microbes, i.e., *Blautia hydrogenotrophica*, was observed. *Blautia hydrogenotrophica*, *Bifidobacterium pseudocatenulatum*, uncharacterized *Blautia* CAG:257 and uncharacterized *Actinomyces* ICM7 (Q = 0.006, 5.6 × 10^−5^, 0.001, 0.02). In addition, the feces of MBD subjects had higher levels of total SCFA (*p* = 0.001), acetate (*p* = 0.002), propionate (*p* = 0.007) and butyrate (*p* = 0.0005) than those of WD subjects [69].

For comparison, the Mediterranean diet, based mainly on cereals, fruits and vegetables, vegetable oils, especially olive oil, legumes, nuts, and limited amounts of fish and poultry, has also been examined in relation to gut microbiota composition. This dietary pattern is characterized by low consumption of red meat and meat products, dairy products, and sweets. Some studies reported that higher adherence to the Mediterranean diet was associated with higher fecal SCFA levels and changes in selected bacterial taxa, including *Prevotella* and *Firmicutes* [72].

However, Kimble et al. [72], in a systematic review of 17 RCTs and 17 observational studies, explored the effects of the Mediterranean diet on gut microbiota composition and microbial metabolism. They found that although some studies indicated a positive effect of the Mediterranean diet on selected gut microbial taxa, the overall results did not consistently show changes in gut microbiota composition or microbial metabolism. As the authors emphasized, this may be related to heterogeneity between the studied populations, analytical methods, intervention duration, and characteristics of the Mediterranean diet [72].

### 4.1. Protein

As a basic building block, protein is essential for the development and daily functioning of the human body. A distinction can be made between proteins of plant and animal origin. Plant protein sources include pulses, cereals, and nuts. Protein of animal origin, which by far predominates in Western-type diets in many populations, is found in the form of meat, dairy, eggs, and products derived from them [73].

In a study on the effect of proteins of various origins on the quality of microorganisms residing in the colon, whey protein and pea protein were found to have a beneficial effect on the diversity of microorganisms in the intestine. An increase in the commensal bacteria *Bifidobacterium* and *Lactobacillus* was demonstrated. In addition, the group consuming whey protein showed reduced concentrations of pathogenic *Clostridium perfringens* and *Bacteroides fragilis*. In contrast, pea protein increased the concentration of beneficial SCFAs in the intestinal lumen [74,75]. In contrast, a study of animal protein consumption showed an increased number of bacteria resistant to high bile concentrations. These included *Bacteroides*, *Alistipes*, and *Bilophila* [70]. Analysis of fecal levels of short-chain fatty acids (SCFAs) and selected inflammatory markers in serum provides a better understanding of the effects of diet on the gut microbiota and inflammation in the body. A study comparing a low-carbohydrate diet (LCD) with a classical diet (CLD) in obese women following a calorie-restricted diet showed significant differences in SCFA and inflammatory marker levels. The CLD group showed a significant increase in interleukin-6 (IL-6) levels (*p* < 0.001), which may indicate increased inflammation. After adjusting for baseline values, fecal butyric acid, propionic acid and acetic acid levels were significantly different between the LCD and CLD groups (*p* < 0.001, *p* = 0.02 and *p* < 0.001, respectively). Importantly, an increase in serum insulin levels correlated with a decrease in fecal propionic acid levels by as much as 5.3-fold (95% CI = −2.7, −0.15, *p* = 0.04). In addition, increased levels of high-sensitivity C-reactive protein (hs-CRP) were associated with a 25% reduction in the proportion of butyric acid in feces (*p* = 0.04), which may suggest a link between inflammation and SCFA metabolism. Fasting glucose (FBS) and insulin concentrations significantly affected acetic acid levels (*p* = 0.009 and *p* = 0.01, respectively). Higher FBS and insulin values were associated with a 2.8- and 8.9-fold increase in fecal acetic acid levels (95% CI = 0.34) [76].

In addition, high intake of red meat may increase the availability of L-carnitine, which can be metabolized by gut microbiota to trimethylamine and subsequently oxidized to trimethylamine N-oxide (TMAO), a metabolite associated with atherosclerosis and cardiovascular risk [7,77].

The amount of protein consumed is also a factor that significantly affects the composition of the gut microbiota. A 2021 randomized clinical trial [78] involving 50 participants showed that after just 1 week of consuming ~1.6 g protein/kg m. c/day in stool samples, there was a significant decrease in *Veillonellaceae Veillonella* (log_2_ FC = 21.90, *p* < 0.001), *Veillonellaceae Megasphaera* (log_2_ FC = 22, 11, *p* < 0.001), *Akkermansiaceae Akkermansia* (log_2_ FC = 3.09, *p* = 0.04), *Eggerthellaceae* (log_2_ FC = 24.84, *p* < 0.001) and *Ruminococcaceae* (log_2_ FC = 5.84, *p* = 0.01) [78]. In addition, a 2021 meta-analysis by Hsu et al. [79] including five studies, including four clinical trials with a control group and one prospective observational study, showed that the use of a low-protein diet (<0.8 g/kg m. c/dz) in patients with chronic kidney disease leads to significant changes in their intestinal microbiota including an increase in *Lactobacillaceae* (*p* = 0.010), *Bacteroidaceae* (*p* = 0.048), *Streptococcus anginosus* (*p* < 0.001) and a decrease in *Bacteroides eggerthii* (*p* = 0.017) and *Roseburia faecis* (*p* = 0.019) compared with patients on a normoprotein diet. At the same time, the study authors found no significant changes in the diversity of α and β gut microbiota of patients on a low-protein diet [79].

In contrast, a 2022 systematic review by Wu et al. [80] covering 29 studies, including 6 studies in mice, 7 studies in pigs, 15 studies in rats, and 1 in vitro study showed that it is not only the amount and type of protein consumed that have a significant impact on the composition and activity of the intestinal microbiota but also the processing techniques play an important role. Food processing technologies, depending on the chosen method, can cause several structural or microstructural modifications in proteins, thereby leading to changes in their digestibility and functional properties, which translate into the availability of the nutrient to the intestinal microbiota and how it is utilized. Two chemical reactions occurring during food processing and preparation processes—glycation and oxidation—appear to play a particular role in the variability of the effect of proteins on the diversity of the intestinal microbiota. However, with new technological methods emerging, further research in this area is needed [80]. At this point, the detailed link between proteins and the gut microbiome has not yet been thoroughly studied, which also results in a small amount of research on this topic.

### 4.2. Fat

Fat in the human diet, depending on its composition and quantity, can act preventively or increase the risk of many diseases, such as cardiovascular disease. Monounsaturated and polyunsaturated fatty acids have a preventive effect against most diseases of civilization. Saturated and trans fatty acids increase total cholesterol and LDL fractions and have pro-inflammatory effects, so that when consumed in inappropriate amounts, they can contribute to diseases such as obesity, metabolic syndrome, type 2 diabetes, and also neurological and cognitive disorders [81].

A study was conducted with 217 healthy adults on the amount of fat consumed and its effect on gut bacteria composition [82]. They were divided into 3 groups with isocaloric diets of varying fat content. There was a group with a low-fat content (20% of dietary energy), a moderate fat content (30% of energy), and a high fat content (40% of energy). It was shown that the low-fat diet was associated with an increase in microbiota diversity, including an increase in butyrate-producing bacteria such as *Blautia* and *Faecalibacterium*. In contrast, the high-fat diet group showed reduced SCFAs, as well as increased excretion of arachidonic acid with feces [82].

It was also investigated how the consumption of farmed salmon, a rich source of monounsaturated and polyunsaturated acids, would affect the composition and activity of the microbiota of 123 pregnant women who were participants in the study [83]. This study, however, showed no significant differences in the microbiota composition of both the mother and the baby from whom fecal samples were collected after delivery. Nevertheless, this study showed that adequate maternal weight gain during pregnancy was associated with increased abundance of gut bacteria, including *Bifidobacterium* and *F. prausnitzii* [83].

In a study that compared the effects of fatty acids from fish oil and lard, it was found that the group of mice consuming lard had a higher percentage of *Bacteroides* and *Bilophila*, while the group consuming fish oil had a higher percentage of lactic acid bacteria (*Lactobacillus* and *Streptococcus*), *Verrucomicrobia* (*Akkermansia muciniphila*) and *Actinobacterium* (*Bifidobacterium* and *Adlercreutzia*). Also highly significant were an increase in Toll-like receptors (TLR) activity, a decrease in insulin sensitivity and the occurrence of inflammation in white adipose tissue (WAT) in lard-treated mice. The authors of the study concluded that a diet with a predominance of saturated fatty acids can induce inflammation and metabolic dysfunction in the body. This is due to excessive TLR activation, which is at least partially correlated with the occurrence of changes in the gut microbiota as a result of a diet rich in saturated fatty acids [84].

A randomized controlled crossover study published in 2023 [85] showed that replacing the intake of SFA (saturated fatty acids) containing products with products high in PUFA (polyunsaturated fatty acids) leads to an increase in the abundance of *Lachnospiraceae* (*p* = 0.013) and *Bifidobacterium* spp. (*p* = 0.029). In addition, consumption of polyunsaturated fatty acids (PUFAs) for three consecutive days led to an increase in the abundance of *Alistipes* (*p* = 0.006), *Parabacteroides johnsonii* (*p* = 0.014) and *Lachnospiraceae* (*p* = 0.009), and increased the relative level of butyrate in the blood (*p* = 0.015). Interestingly, the increase in *Lachnospiraceae* caused by changing the quality of dietary fats correlated with a decrease in total cholesterol, a promising finding for the prevention and treatment of hypercholesterolemia [85].

A 2019 meta-analysis by Bisanz et al. [86] involving 25 studies in mouse models found a significant and reproducible effect of a high-fat diet (27.1–65% kCal) on gut microbiota structure. The high-fat diet caused a significant decrease in gut bacterial richness by Chao1 index (log_2_ FC = −0.215, 95% CI −0.270 to −0.159, *p* = 8.73 × 10^−14^), Shannon diversity (log_2_ FC = −0.048, 95% CI −0.084 to −0.013, *p* = 7.83 × 10^−3^) and Faith phylogenetic diversity (log_2_ FC = −0.122, 95% CI −0.157 to −0.088, *p* = 7.26 × 10^−12^). In addition, it caused an increase in the ratio of *Firmicutes* to *Bacteroidetes* (log_2_ FC = 1.84, 95% CI 1.65 to 2.03, *p* = 3.4 × 10^−69^) [86].

### 4.3. Carbohydrates

Carbohydrates, which are the primary source of energy for most of the body’s cells, are also the best-studied food component in terms of their effects on the microbiota. The basic division of carbohydrates distinguishes between assimilable and non-assimilable carbohydrates. Assimilable carbohydrates are digested in the small intestine with the help of enzymes. These include sugars such as lactose, sucrose, glucose and fructose, as well as starch. Once these components are broken down and absorbed into the bloodstream, the released glucose activates the pancreas for an insulin response [87]. In contrast, non-absorbable carbohydrates, which include resistant starch and fiber, are not digested by enzymes in the upper gastrointestinal tract. They mostly pass into the large intestine, where they are fermented by bacteria residing in the distal part of the intestine [87].

High intake of simple sugars may disturb gut microbiota balance and promote a more pro-inflammatory microbial profile. Previous evidence suggests that high sugar intake may increase the relative abundance of *Proteobacteria* and decrease *Bacteroidetes*, although the direction of changes may depend on the type of sugar, dose, and study model [88]. A study conducted in 2023 by a team of Japanese researchers used a multiomics strategy, combining fecal metabolomics, metagenomics, host metabolomics and transcriptomics to determine the impact of the microbiome on insulin resistance. The results indicate that people with insulin resistance have an increased number of monosaccharides available to the body, which is associated with bacterial carbohydrate metabolism and inflammation. The identified gut bacteria vary in carbohydrate metabolism, and those associated with insulin sensitivity may alleviate insulin resistance, suggesting potential therapeutic targets [89].

Wang et al. [90] conducted an experiment to evaluate changes in the structure and function of intestinal microbiota induced by a fructose-rich diet. They found that high fructose intake (10.5 g/kg/day) for 20 weeks led to an increase in the abundance of *Lachnospira*, *Parasutterella*, *Marvinbryantia* and *Blantia* in the large intestine of rats [90]. Separately, the effect of adding lactose to the previously mentioned sugars was also studied. Separately, the effect of lactose was also studied. Lactose, which may be poorly tolerated by lactase non-persistent individuals, has been shown to modify fecal microbiota composition, including changes in selected *Clostridia* groups and SCFA concentrations [91]. An in vitro study conducted by Firrman et al. [92] showed that lactose, by reducing the relative abundance of *Bacteroidaceae* and increasing the abundance of *Lactobacillaceae*, *Enterococcaceae*, *Streptococcaceae*, and *Bifidobacterium*, modifies the structure of the gut microbiota. These changes were accompanied by an increase in the total amount of SCFAs, particularly acetate and lactate [92].

In terms of non-absorbable carbohydrates, the results of all studies were unequivocal, suggesting that increased fiber intake promotes microbiota diversity. Dietary fiber is the part of the cell wall of plants, algae, or fungi that is resistant to hydrolysis occurring under the influence of digestive enzymes. It is distinguished from other prebiotics by the fact that it can be utilized by most of the microorganisms that populate the colon and not just by selected strains of bacteria. Fiber is divided into two fractions—water-soluble and water-insoluble. Each of them, despite their different functions, has beneficial effects on the body. What is important, however, is their amount in the diet and their origin. However, excess fiber can cause digestive problems and disrupt the rhythm and number of bowel movements [93].

A meta-analysis conducted by Stachowska et al. [94] showed that dietary fiber intake significantly affected parameters such as ALT levels (SMD = −0.667; 95% CI −1.046 to −0.288, *p* = 0.001), AST (SMD = −0.466; 95% CI −0.840 to −0.091, *p* = 0.015), HOMA-IR (SMD = −0.619; 95% CI −1.026 to −0.211, *p* = 0.003), insulin (SMD = −0.705; 95% CI −1.115 to −0.295, *p* = 0.001 and BMI (SMD = −0.494; 95%CI −0.864 to −0.125, *p* = 0.009 subjects. A decrease in the above parameters is associated with a reduced risk of NAFLD, insulin resistance, and obesity, which also validates the conclusions about the validity of introducing more prebiotic products into the diet both in prevention and in individuals at risk of the above disease entities [94].

A 2018 meta-analysis by So et al. [95] involving a total of 58 randomized clinical trials found that the use of dietary fiber (1.2 g/day to 50 g/day) in healthy adults for 5 days to 3 months compared with placebo or a low-fiber comparison group increased the abundance of *Bifidobacterium* spp. (standardized mean difference (SMD = 0.64; 95% CI: 0.42–0.86; *p* < 0.00001; *I*^2^ = 85% *p* < 0.00001) and *Lactobacillus* spp. (SMD = 0.22; 95% CI: 0.03–0.41; *p* = 0.02; *I*^2^ = 49%, *p* < 0.005) in the intestinal microbiota and an increase in fecal butyrate (SMD = 0.24; 95% CI: 0.00–0.47; *p* = 0.05; *I*^2^ = 70%, *p* < 0.00001). In addition, subgroup analysis showed that supplementation with fructans and galactooligosaccharides resulted in significantly higher levels of *Bifidobacterium* spp. (SMD = 0.68; 95% CI: 0.38–0.98; *p* < 0.00001, *I*^2^ = 81%, *p* < 0.00001) compared with a low-fiber placebo [95].

Results of a 2021 meta-analysis [96] aimed at evaluating, among other things, the effect of dietary fiber on intestinal microbiota in patients with type 2 diabetes also show a significant increase in the relative abundance of *Bifidobacterium* (MD = 0.73, 95% CI: 0.57–0.89; *p* < 0.05; *I*^2^ = 77%, *p* < 0.01) in the group receiving dietary fiber compared with the control group [96].

### 4.4. Low-Calorie Sweeteners

The effect on the microbiota of artificial sweeteners is also an important issue. Although these substances were initially portrayed as health-promoting, it has been shown that they can reduce glucose and sucrose tolerance by affecting bacteria residing in the colon. Saccharin administration in mice was associated with gut dysbiosis, including an increased abundance of *Bacteroides* and a reduced abundance of *Lactobacillus reuteri* [87,97]. A 2024 meta-analysis by Chen et al. [98] involving a total of nine studies, including seven in animal models and two involving humans, showed a significant effect of sucralose consumption on gut microbiota composition in both mice/rats and humans. In rodents, sucralose consumption compared with the control group induced a statistically significant decrease in the relative abundance of *Bacteroidetes* by 11.02 (95% CI, −19.68 to −2.37, *p* = 0.01; *I*^2^ = 99%, *p* < 0.01), *Firmicutes* by 2.88 (95% CI, −11.80 to 6.05, *p* = 0.53; *I*^2^ = 98%, *p* < 0.01), *Proteobacteria* by 1.52 (95% CI, −4.27 to 1.22, *p* = 0.28; *I*^2^ = 99%, *p* < 0.01) and *Verrucomicrobia* by 1.43 (95% CI, −2.80 to −0.07, *p* = 0.04; *I*^2^ = 95%, *p* < 0.01) and an increase in the relative abundance of *Actinobacteria* by 0.0006 (95% CI, −0.28 to 0.28, *p* = 0.99; *I*^2^ = 75%, *p* < 0.01). As a consequence, the present changes in the test animals were observed in the development of obesity-related diseases. However, in humans, the relative abundance of *Bacteroidetes* significantly increased by 0.24 (95% CI, 0.10 to 0.39, *p* < 0.001, compared with the control group) as a result of sucralose consumption; *I*^2^ = 1%, *p* = 0.32), *Firmicutes* by 0.02 (95% CI, −0.08 to 0.11, *p* = 0.73; *I*^2^ = 0%, *p* = 0.34), and *Actinobacteria* by 0.30 (95% CI, 0.17 to 0.42, *p* < 0.01; *I*^2^ = 42%, *p* = 0.19), resulting in reduced obesity. The gut microbiota alpha diversity according to the Shannon index in rodents decreased by 0.12 (95% CI, −0.48 to 0.24, *p* = 0.52; *I*^2^ = 0%, *p* = 0.54), while in humans it increased by 0.09 (95% CI, −0.10 to 0.28, *p* = 0.36; *I*^2^ = 0%, *p* = 0.54), but both changes were statistically insignificant [98].

### 4.5. Polyphenols

Polyphenols are a group of compounds that include anthocyanins, proanthocyanidins, catechins, flavones, flavonols, and phenolic acids. Their main sources are vegetables and fruits (especially berries), as well as cocoa, tea and wine. These are substances that exhibit antioxidant activity. Consuming a lot of products rich in polyphenols leads to an increase in the number of *Bacteroides*, *Bifidobacterium*, and *Lactobacillus* (mainly after drinking red wine) [99]. A study evaluating the effect of polyphenols found in fruits showed that the pathogenic bacteria *Staphylococcus aureus* and *Salmonella typhimurium* are highly sensitive to the antimicrobial properties of polyphenols. Consumption of large amounts of polyphenols also reduces the pathogenic activity of *Clostridium bacteria* [100]. The results of a 2020 meta-analysis [101] including 27 studies show that supplementation with polyphenols from various sources viz. apples, tea, wine, coffee or soy significantly increased *Lactobacillus abundance* (in vivo, SMD = 0.23, 95% CI 0.04–0.42, *I*^2^ = 42%, *p* = 0.08; in vitro, SMD 5.02, 95% CI 2.49–7.55; *I*^2^ = 98%, *p* < 0.01) and *Bifidobacterium* (in vivo, SMD 0.56, 95% CI 0.23–0.89, *p* < 0.01; *I*^2^ = 83% *p* < 0.01; in vitro, SMD 4.29, 95% CI 1.83–6.74, *p* < 0.01; *I*^2^ = 99%, *p* < 0.01) in the gut microbiota. It was also noted that the peak increase in the abundance of these bacteria followed the consumption of polyphenols at doses of 396 mg/d, 540 mg/d, and 593 mg/d [101].

### 4.6. Probiotic Foods

Foods containing probiotics are classified as functional foods. This is a group of food products of natural origin that have been so modified or subjected to such technological treatments as to carry additional positive health effects for humans. Such effects may include reducing the growth of putrefactive bacteria, improving intestinal peristalsis, and increasing the production of digestive juices. Probiotic products can also lead to a reduced incidence of diarrhea and attenuation of symptoms of lactose intolerance [102].

Probiotic foods mainly include fermented dairy products and pickles, which are fermented plant products. During the fermentation of such products, the proliferation of probiotic bacteria and fungi takes place. However, this proliferation should be controlled so that human pathogenic strains do not develop [102,103].

Until a decade or so ago, the most popular dairy probiotic product was fermented (curdled) milk; currently, however, we have a much larger selection of dairy products to support the human microbiota, such as kefir and yogurt. In addition, nowadays much more attention is paid to the selection of strains used in the production of functional foods so that they also meet the criteria for probiotic strains [102,104]. These criteria include sufficient characterization of the strain in question; safety during proper use, supported by at least one clinical study conducted on humans under accepted standards, the result of which was positive; and stability of the strain, giving confidence that its dose in the medicinal product will be the same throughout its shelf life [104]. For a strain to qualify as a probiotic strain, it must meet the following requirements:

1. The strain should be thoroughly characterized.
2. It is safe when used as directed.
3. It should be supported by at least one human clinical study with positive results.
4. Shall be present in a dose that demonstrates efficacy in specific products throughout their shelf life.

If any of the above guidelines are not met, the strain cannot be called a probiotic strain and its deliberate addition to food should not be used [104].

In addition to the dairy products known by all, it is possible to distinguish a much broader group of products that, through the fermentation that takes place, provide various strains of probiotics—not only LAB. At the same time, it should be noted that foods in which the composition of bacteria has not been precisely determined cannot be called probiotic products [105]. Products of plant origin with probiotic properties that can be found on store shelves are shown in Table 4.

The selection of plant products containing probiotic bacteria is sizable, but there have not yet been a sufficient number of clinical studies proving their unequivocal effect on the state of the intestinal microbiota.

Milk and fermented dairy products, in addition to their probiotic value, are also rich in fat-soluble vitamins, calcium, protein, and phosphorus. The most common fermented dairy products are described in Table 5.

Unfortunately, food products containing probiotics do not have the same microbiological stability as pharmaceutical preparations. This is due, among other things, to the environment and time of their storage, as well as the heat treatment they may undergo [116]. The content of probiotic bacteria in the product is also affected by the oxidative stress that occurs, the pH that is too low, inappropriate starter cultures, storage temperature, and the type of technological processes to which the products are subjected. Therefore, it is important to remember that while fermented foods are a good addition to the daily diet, they will not always work well in the case of disturbed intestinal microbiota [116]. Each fermented product may have a different content of probiotic bacteria, which can reach and populate the large intestine to varying degrees. Thus, in the case of antibiotic therapy or other intestinal microbiota disorders, probiotic pharmacological therapy should be considered, which can be supported by functional foods [116].

In recent years, studies have been conducted on the effectiveness of dairy products for the prevention of infections occurring outside the gastrointestinal tract. They indicated that regular consumption of fermented dairy may have a much broader application than just modulating the intestinal microbiota. Higher intake of dairy (especially reduced-fat dairy) may be associated with a reduced risk of type 2 diabetes, hypertension, overweight and obesity [113,114,117,118].

A randomized controlled trial conducted with 60 healthy overweight individuals evaluated the effect of probiotic supplementation with VSL#3, a multi-strain probiotic preparation containing one strain of *Streptococcus*, four strains of *Lactobacillus*, and three strains of *Bifidobacteria* [119]. The results show that the total amount of aerobic bacteria in the intestine changed under the influence of the supplementation. The content of anaerobic bacteria *Lactobacillus*, *Bifidobacteria*, and *Streptococcus* also increased in the group that did not take placebo products. The group consuming probiotic dairy also had significantly reduced levels of *E. coli* bacteria. Triglycerides, total cholesterol, and LDL fraction, as well as C-reactive protein levels also decreased. In a separate study, probiotics-containing yogurt ingestion combined with *H. pylori* eradication was associated with restoration of fecal *Faecalibacterium prausnitzii* dysbiosis in *H. pylori*-infected children [120].

## 5. Drugs and Dietary Supplements to Support the Gut Microbiota

Currently, a great number of pro-, pre-, and symbiotic preparations are available on the market. Three groups can be distinguished [121]:

- Medicinal products—Substances or mixtures of substances that exhibit a curative or preventive effect against an existing disease;
- Dietary supplements—Foodstuffs supplementing the daily diet, being rich in vitamins, minerals, or other substances having a nutritional effect on the body. Their form must allow free dosage;
- Foods for special medical purposes—Foods modified in such a way that their composition meets the nutritional needs of patients. Used as the sole source of energy supply or as an adjunctive therapy in patients who cannot supply themselves with all the necessary nutrients.

## 6. Discussion

The proper state of the gut microbiota is extremely important to maintain the body’s homeostasis and reduce the risk of many chronic diseases such as type 2 diabetes, obesity, allergies, cardiovascular, gastrointestinal, and neurodegenerative diseases. The diversity and number of microorganisms in the intestinal lumen depend on a variety of factors. These include the type of delivery, past infections and the amount of antibiotics used, lifestyle, and type of diet [122].

A diet rich in prebiotic compounds prevents dysbiosis in the large intestine. However, if there is an imbalance between probiotic and putrefactive bacteria, a properly managed diet, which, if necessary, is enriched with supplementation of probiotic or prebiotic products, can improve the gut microbiota after just a few weeks. Saccharides that stimulate the growth of gut bacteria may contribute to reducing the risk of lifestyle diseases [123,124,125].

Prebiotics selectively stimulate the growth and activity of beneficial gut bacteria, particularly *Bifidobacterium* and *Lactobacillus*, leading to improved microbiota balance and host health [10]. Due to their fermentation by gut microbiota, short-chain fatty acids (SCFAs) such as butyrate, acetate, and propionate are formed, which serve as an energy source for enterocytes and regulate inflammation [10,11]. In turn, probiotics compete with pathogens for attachment sites in the intestine and for nutrients, which reduces the risk of colonization by harmful bacteria. They produce antimicrobial compounds, such as carbon dioxide, hydrogen peroxide, acetaldehyde, acetoin, diacetyl, bacteriocins and bacteriocin-like inhibitory substances, which inhibit pathogen growth [11]. Probiotics, modulating the intestinal microbiota, trigger the expression of genes responsible for the modification and maturation of the intestine after birth, absorption of nutrients, strengthening the mucosal barrier, metabolism of xenobiotics and angiogenesis [10,11]. By strengthening the intestinal barrier, gut permeability is reduced, which prevents translocation of pathogenic bacteria and pro-inflammatory molecules [11].

After thorough analyses, which were performed on the participants of the study conducted by David et al. [68] during each day of the experiment, as well as after its completion, the researchers came to the unequivocal conclusion that the microbiota can undergo dynamic changes under the influence of introduced changes in the human diet. At the same time, it was proven that while a plant-based diet did not affect the deterioration of the microbiota, a diet full of animal products significantly disrupted microbial function in the large intestine [68]. However, it has been suggested that a diet high in undigestible carbohydrates has the strongest effect on increasing the percentage of lactic acid bacteria and bifidobacteria in the intestinal lumen. It is worth noting, however, that the group of compounds with prebiotic effects is wide, and each of them has an effect directed toward a specific strain and type of bacteria. Therefore, it is important to diversify the daily diet with all products rich in prebiotic compounds [122,123].

## 7. Conclusions

The available evidence indicates that diet, prebiotics, probiotics, synbiotics, and postbiotics may contribute to the modulation of gut microbiota composition and activity. These strategies may influence intestinal barrier integrity, short-chain fatty acid production, immune regulation, and selected metabolic processes. However, their effects are not uniform and depend on multiple factors, including baseline microbiota composition, health status, dietary pattern, probiotic strain, dose, and duration of intervention. Therefore, microbiota-targeted dietary and supplementation strategies should be interpreted as context-dependent approaches rather than universally effective interventions. Future well-designed clinical studies are needed to better define strain-specific, substrate-specific, and population-specific responses and to support the development of more personalized nutritional strategies.

## 8. Perspectives

While significant progress has been made in understanding the relationship between diet, prebiotics, probiotics, and gut microbiota, several areas require further research. Future studies should explore the most effective combinations of prebiotics and probiotics for targeting specific health conditions. More research is needed to understand how individual differences in microbiota composition influence responses to dietary interventions. Clinical trials should investigate the prolonged impact of prebiotic and probiotic supplementation on metabolic health, immune function, and disease prevention. Additionally, further examination of non-carbohydrate prebiotics, such as polyphenols and certain fatty acids, is necessary to determine their potential role in modulating gut microbiota. The development of personalized dietary strategies based on microbiota profiles could enhance precision medicine applications in gut health. Addressing these research gaps will allow for a more comprehensive understanding of the gut microbiota’s role in human health and aid in developing more effective dietary and therapeutic interventions.

## Figures and Tables

**Table 1:** Effectiveness of probiotic intake depending on doses and duration of supplementation used in various disease entities.

Used Strain/Strains	Intake duration	Dose	Type of Preparation	Cohort	Impact on Parameters	Impact on Organism	Type of Study	Reference
*Lactobacillus casei* Shirota 10^8^/mL	1 week	130 mL (65 mL twice daily)	Commercial strain microbial cell preparation	142 hospitalized patients with acute gastroenteritis	↓ hs-CRP *, WBC *, ↑ eGFR *	Reduction in leukocyte, inflammation, frequency of bowel movement, improvement in renal parameter	A hospital-based clinical study	Akoglu et al., 2015 [35]
Probiotic soy milk containing *Lactobacillus plantarum* A7 (2 × 10^7^ CFU/mL)	8 weeks	200 mL/day	Single strain	48 T2DM-DN patients (case 24 and control 24)	↓ Serum NGAL, Cys-c *, ↑ sTNFR1 (ng/mL)	Enhanced renal function	Parallel RCT	Miraghajani et al., 2018 [36,37]
Probiotic soy milk containing *Lactobacillus plantarum* A7 (2 × 10^7^ CFU/mL)	8 weeks	200 mL/day	Single strain	44 T2DM-DN patients from Iran (case 22 and control 22)	↓ sCr (mg/dL) ^, ↑ eGFR ^	Enhanced glomerular function	Double-blind RCT	Abbasi et al., 2017 [38]
Probiotic Kefir *Streptococcus thermophiles*, *Lactobacillus casei*, *Lactobacillus acidophilus*, and *Bifidobacterium lactis*	8 weeks	600 mL twice/day	Multi-strain microbial cell preparation	60 T2DM (case 30 and control 30)	↓ HbA1c ^, TG, TC	Enhanced glycaemic control	Randomized double-blind placebo-controlled clinical trial	Ostadrahimi et al., 2015 [39]
<4 × 10^9^ CFU/day mixed culture probiotics	6, 8, and 12 weeks	Microbial cell preparation, probiotic soy milk, probiotic capsule, and tablet	Single and multi-strain microbial cell preparation	10 RCTs (552 patients)	↓ sCr ^#^, Cys-c +, urinary Alb/Cr ^#^, serum Na, BUN ^	Enhanced inflammation and oxidative stress, improved kidney, lipid, and glucose biomarkers	Systematic review and meta-analysis	Dai et al., 2022 [40]
*Lactobacillus plantarum* A7, *Bacillus coagulans* T11 > 5 billion CFU	8 and 12 weeks	Probiotic soy milk, capsule, sachet, honey	Single- and multi-strain microbial cell preparation	7 RCTs (220 patients)	↓ MDA, hs-CRP ^, ↑ GSH ^#^, TAC	Enhanced renal function and glucose control	A systematic review and meta-analysis of clinical trials	Bohlouli et al., 2021 [41]
Supplemental form in capsule *Lactobacillus acidophilus* strain ZT-L1, *Bifidobacterium bifidum* strain ZT-B1, *Lactobacillus reuteri* strain ZT-Lre, and *Lactobacillus fermentum* strain ZT-L3 (each strain dose: 2 × 10^9^) total: 8 × 10^9^ CFU/day	12 weeks	One capsule/day	Multi-strain microbial cell preparation	60 T2DM-DN (case 30 and control 30)	↓ FBG, HOMA-IR ^#^, hs-CRP ^, MDA ^, ↑ insulin sensitivity, HDL-c ^, GSH ^	Enhanced glucose control, reduced cardiac risk, inflammation	Placebo-controlled RCT	Mafi et al., 2018 [42]
Probiotic honey *Bacillus coagulans* T11 (IBRC-M10791) (10^8^ CFU/g)	12 weeks	25 g/day	Viable and heat-resistant probiotic	60 patients with DN (case 30 and control 30)	↓ serum insulin ^#^, HOMA-IR ^, hs-CRP, MDA ^, ↑ total-/HDL-cholesterol	No significant enhancement in the metabolic profiles	Randomized, double-blind, controlled clinical trial	Mazruei Arani et al., 2019 [43]
*Lactobacillus acidophilus*, *Lactobacillus casei*, *Lactobacillus lactis*, *Bifidobacterium bifidum*, *Bifidobacterium longum*, and *Bifidobacterium infantis* (6.0 × 10^10^ CFU/day total) mixed in water	12 weeks	Two sachets/day	Freeze-dried multi-strain microbial cell preparation	136 T2DM (case 68 and control 68)	↓ BUN *	Enhanced renal function	Double-blind, parallel RCT	Firouzi et al., 2015 [44]
*Bifidobacterium bifidum*, 1.2 × 10^9^ CFU, *Lactobacillus acidophilus* 4.2 × 10^9^ CFU, *Streptococcus thermophilus* 4.3 × 10^9^ CFU/day	12 weeks	One capsule/day	Multi-strain microbial cell preparation	76 T2DM (case 34 andcontrol 42)	↓ urinary Alb/Cr (mg/g) *, ↑ HbA1C *, FBG *	Enhanced renal function and glucose control	Double-blind, placebo-controlled RCT	Jiang et al., 2021 [45]
*L. acidophilus*, *B. bifidum*, and *Bifidobacterium longum*	12 weeks	(200 mg/day) + (2 × 10^9^ CFU/day each)	selenium + multi-strain probiotic preparation	90 AD (27 cases, 26 cases with only selenium, and 26 control)	↑ TAC ^ and GSH ^ level, ↓ serum hs-CRP ^	Enhanced cognitive function, reduction in biomarkers of inflammation and oxidative stress, metabolic status and gene expression related to inflammation, insulin, and lipid	Double-blind, RCT	Tamtaji et al., [46]

↓: decrease; ↑: increase; RCT: randomized clinical trial; T2DM: type 2 diabetes mellitus; CFU: colony forming units; BUN: blood urea nitrogen; hs-CRP: high-sensitivity C-reactive protein; WBC: white blood cell; eGFR: estimated glomerular filtration rate; HbA1c: glycated hemoglobin; TG: triglyceride; TC: total cholesterol; T2DM-DN: type 2 diabetes mellitus with diabetic nephropathy; sCr: serum creatinine; NGAL: neutrophil gelatinase-associated lipocalin; sTNFR1: soluble tumor necrosis factor receptor 1; FBG: fasting blood glucose; HOMA-IR: homeostatic model assessment for insulin resistance; MDA: malondialdehyde; GSH: glutathione; HDL-c: high density lipoprotein; urinary Alb/Cr: urinary albumin creatinine ratio; TAC: total antioxidant capacity; Na: sodium; * statistical significant *p* < 0.05; ^ statistical significant *p* < 0.001; ^#^ statistical significant *p* < 0.004; + statistical significant *p* < 0.00001.

**Table 2:** Effects of probiotic therapy on selected parameters in sick and healthy subjects [47].

	Probiotic	Control	Effect Size
Body Mass Index [47]	*L. acidophilus*, *L. casei*, *L. lactis*, *Bifdobacterium*, *Actinobacteria*, *B. bifdum*, *B. longum*, *B. infantis*, *L. rhamnosus* HN001, *L. bulgaricus*, *L. salivarius* W24, *L. sporogenes*, *Bifidobacterium breve*, *Streptococcus thermophilus*, *L. helveticus* Cardi04	Maltodextrin/Placebo/Maize starch and maltodextrins/Fructo-oligosaccharide, and Magnesium stearate/Isomalt, sorbitol and stevia/Control food/Artificially acidified milk/Lactose and magnesium stearate	SMD ^(a,d)^ = −0.42, 95% CI (−0.76;−0.08); *I*^2^ = 89.6%, *p* = 0.000
	*L. acidophilus*, *L. casei*, *L. lactis*, *Bifdobacterium*, *Actinobacteria*, *B. bifdum*, *B. longum*, and *B. infantis*, *B. bifdum* W23, *B. lactis* W52, *L. acidophilus* W37, *L. brevis* W63, *L. casei* W56, *L. salivarius* W24, *L. lactis* W19, and *L. lactis* W58	placebo/Maize starch and maltodextrins	SMD ^(b,d)^ = −0.62, 95% CI (−1.13;−0.11); *I*^2^ = 90.6%, *p* = 0.000
	*L. casei*/*L. sporogenes*/*L. helveticus* Cardi04/*Bifidobacterium*/*L. reuteri* DSM 17938/*Brewer’s yeast*/*L. rhamnosus* HN001/*Lactiplantibacillus plantarum* OLL2712	Maltodextrin/Isomalt, sorbitol and stevia/Control food/Artificially acidified milk/Lactose and magnesium stearate/placebo/Cellulose microcrystalline compounds, magnesium stearate, caramel, malt, and stearic acid/Microcrystalline cellulose and dextrose anhydrate	SMD ^(c,d)^ = −0.21, 95% CI (−0.70; 0.27); *I*^2^ = 89.6%, *p* = 0.000
Fasting Blood Glucose Concentration [47]	as below	as below	SMD ^(a,d)^ = −0.73, 95% CI (−0.97; −0.48); *I*^2^ = 89.5%, *p* = 0.000
	*L. acidophilus*, *L. casei*, *L. lactis*, *Bifdobacterium*, *Actinobacteria*, *B. bifdum*, *B. longum*, and *B. infantis*, *B. bifidum* W23, *B. lactis* W52, *L. acidophilus* W37, *L. brevis* W63, *L. casei* W56, *L. salivarius* W24, *L. lactis* W19, *L. lactis* W58, *L. rhamnosus*, *L. bulgaricus*, *B. breve*, *B. longum*, *Streptococcus thermophilus*, *L. bifidum*, *L. family*, *B. family*, *B. lactis* Bb12, *L. acidophilus* La5, *Fermented milk* (kefir containing *L. casei*, *L. acidophilus*, and *Bifidobacteri*), *Lactococcus*, *B. Propionibacterium*, *Acetobacter*, *L. salivarius* UBLS22, *L. casei* UBLC42, *L. plantarum* UBLP40, *L. acidophilus* UBLA34, *B. breve* UBBr01, and *B. coagulans* Unique IS2, *L. paracasei*, *Lacticaseibacillus paracasei* YIT 9029 (strain Shirota:LcS) organisms, *B. breve* YIT 12272, *L. plantarum* Lp-115, *L. bulgaricus* Lb-64, *L. gasseri* Lg-36, *B. breve* Bb-03, *B. animalis* sbsp. lactis Bi-07, *B. bifidum* Bb-06, *Streptococcus thermophilus* St-21, *Saccharomyces boulardii* DBVPG 6763, *Streptococcus thermophilus* (DSM24731), *Bifidobacteria breve* (DSM24732), *Bifidobacteria longum* (DSM2473), *Bifidobacteria infantis* (DSM24737), *L. acidophilus* (DSM24735), *L. plantarum* (DSM24730), *L. paracasei* (DSM24733), *L. delbreuckii* subspecies bulgaricus (DSM24734)	placebo/Maize starch and maltodextrins/Fructo-oligosaccharide, and Magnesium stearate/Magnesium stearate/row starch/Conventional fermented milk/Conventional yogurts/Maize starch and maltodextrins/Fermented milk (dough)/starch/Maltodextrin/corn starch/microcrystalline cellulose, silica, and magnesium stearate	SMD ^(b,d)^ = −0.91, 95% CI (−1.24; −0.57); *I*^2^ = 90.2%, *p* = 0.000
	*L. casei*/*L. sporogenes*/*L. reuteri* DSM 17938/Soy milk containing *L. planetarum*/*L. casei* strain Shirota-fermented milk/*L. helveticus* Cardi04/Brewer’s yeast/*L. paracasei*/*L. plantarum* HAC01/*Bifidobacterium*/OLL2712	Maltodextrin/Isomalt, sorbitol and stevia/Placebo/Conventional soy milk/control bread/Cellulose microcrystalline compounds, magnesium stearate, caramel, malt, and stearic acid/Corn starch/Microcrystalline cellulose/Lactose and magnesium stearate	SMD ^(c,d)^ = −0.44, 95% CI (−0.81; −0.06); *I*^2^ = 88.7%, *p* = 0.000
Fasting Insulin Concentration [47]	as below	as below	SMD ^(a,d)^ = −0.67, 95% CI (−0.99; −0.36); *I*^2^ = 89.4%, *p* = 0.000
	*L. acidophilus*, *L. casei*, *L. lactis*, *Bifdobacterium*, *Actinobacteria*, *B. bifdum*, *B. longum*, and *B. infantis*, *B. bifdum* W23, *B. lactis* W52, *L. acidophilus* W37, *L. brevis* W63, *L. casei* W56, *L. salivarius* W24, *L. lactis* W19, and *L. lactis* W58, *L. rhamnosus*, *L. bulgaricus*, *B. breve*, *B. longum*, *Streptococcus thermophilus*, *L. bifidum*, *L. acidophilus* La-5, *B. lactis* BB-12, *B. lactis* Bb12 and *L. acidophilus* La5, *Lactobacillus*, *Lactococcus*, *Bifidobacterium Propionibacterium*, *Acetobacter*, *L. salivarius* UBLS22, *L. casei* UBLC42, *L. plantarum* UBLP40, *L. acidophilus* UBLA34, *B. breve* UBBr01, and *B. coagulans* Unique IS2,	Placebo/Maize starch and maltodextrins/Fructo-oligosaccharide, and Magnesium stearate/Magnesium stearate/Conventional fermented milk/Conventional yogurts/Maltodextrin/Starch	SMD ^(b,d)^ = −0.73, 95% CI (−1.09, −0.37); *I*^2^ = 87.1%, *p* = 0.000
	*Lacidophilus casei*/*Lactobacillus sporogenes*/L. reuteri DSM 17938/*L. helveticus* Cardi04/*L. plantarum* HAC01,	Maltodextrin/Isomalt, sorbitol and stevia/Control food/Placebo/Artificially acidified milk/Control bread/Microcrystalline cellulose	SMD ^(c,d)^ = −0.55, 95% CI (−1.19, 0.08); *I*^2^ = 92.7%, *p* = 0.000
Triglyceride Concentration [47]	as below	as below	SMD ^(a,d)^ = −0.30, 95% CI (−0.43, −0.17); *I*^2^ = 47%, *p* = 0.005
	*L. acidophilus*, *L. casei*, *L. lactis*, *Bifdobacterium*, *Actinobacteria*, *B. bifdum*, *B. longum*, and *B. infantis*, *B. bifdum* W23, *B. lactis* W52, *L. acidophilus* W37, *L. brevis* W63, *L. casei* W56, *L. salivarius* W24, *L. lactis* W19, and *L. lactis* W58, *Lactobacillus rhamnosus*, *Lactobacillus bulgaricus*, *Bifidobacterium breve*, *Bifidobacterium longum*, *Streptococcus thermophilus*, *L. bifidum*, *Lactobacillus family*, *Bifidobacterium family*, *B. bifdum* W23, *B. lactis* W52, *L. acidophilus* W37, *L. brevis* W63, *L. casei* W56, *L. salivarius* W24, *L. lactis* W19, and *L. lactis* W58, Fermented milk (kefir containing *L. casei*, *L. acidophilus*, and *Bifidobacteri*), *L. salivarius* UBLS22, *L. casei* UBLC42, *L. plantarum* UBLP40, *L. acidophilus* UBLA34, *B. breve* UBBr01, and *B. coagulans* Unique IS2, *Lactobacillus paracasei*, *Lacticaseibacillus paracasei* YIT 9029 (strain Shirota:LcS) organisms, *Bifidobacterium breve* YIT 12272	Placebo/Maize starch and maltodextrins/Fructo-oligosaccharide, and Magnesium stearate/Magnesium stearate/Row starch/Fermented milk (dough)/Starch/Maltodextrin/Corn starch/	SMD ^(b,d)^ = −0.33, 95% CI (−0.49, −0.16); *I*^2^ = 50.8%, *p* = 0.009
	*Lactobacillus sporogenes*/*L. reuteri* DSM 17938/Soy milk containing *L. planetarum*/*Lactobacillus casei* strain Shirota-*fermented milk*/*L. helveticus* Cardi04/*L. paracasei*/*L. rhamnosus* HN001/*Bifidobacterium*	Isomalt, sorbitol and stevia/Placebo/Conventional soy milk/Artificially acidified milk/Corn starch/Microcrystalline cellulose and dextrose anhydrate/Lactose and magnesium stearate	SMD ^(c,d)^ = −0.24, 95% CI (−0.47, −0.02); *I*^2^ = 38.4%, *p* = 0.123
Low-Density Lipoprotein Concentration [47]	as below	as below	SMD ^(a,d)^ = −0.20, 95% CI (−0.37, −0.04); *I*^2^ = 63.9%, *p* = 0.000
	*L. acidophilus*, *L. casei*, *L. lactis*, *Bifdobacterium*, *Actinobacteria*, *B. bifidum*, *B. infantis*, *B. bifdum* W23, *B. lactis* W52, *L. acidophilus* W37, *L. brevis* W63, *L. casei* W56, *L. salivarius* W24, *L. lactis* W19, and *L. lactis* W58, *Lactobacillus rhamnosus*, *Lactobacillus bulgaricus*, *Bifidobacterium breve*, *Bifidobacterium longum*, *Streptococcus thermophilus*, *L. bulgaricus*, *L. bifidum*, *Lactobacillus family*, *Bifidobacterium family*, *L. rhamnosus*, *L. bulgaricus*, *L. acidophilus* La-5 *B. lactis* BB-12, Fermented milk (kefir containing *L. casei*, *L. acidophilus*, and *Bifidobacteri*, *L. acidophilus*, *L. casei*, *L. lactis*, *Bifdobacterium*, *Actinobacteria*, *B. bifdum*, *B. longum*, and *B. infantis*, *L. salivarius* UBLS22, *L. casei* UBLC42, *L. plantarum* UBLP40, *L. acidophilus* UBLA34, *B. breve* UBBr01, and *B. coagulans* Unique IS2, *Lactobacillus paracasei*,	Fructo-oligosaccharide, and Magnesium stearate/Placebo/Maize starch and maltodextrins/Magnesium stearate/Row starch/Conventional fermented milk/Maize starch and maltodextrins/Fermented milk (dough)/Starch/Maltodextrin/Corn starch/	SMD ^(b,d)^ = −0.18, 95% CI (−0.32, −0.05); *I*^2^ = 28.6%, *p* = 0.125
	*Lactobacillus sporogenes*/*L. reuteri* DSM 17938/*L. helveticus* Cardi04/*L. sporogenes*/*L. paracasei*/*L. rhamnosus* HN001/*Bifidobacterium*	Isomalt, sorbitol and stevia/Placebo/Artificially acidified milk/Control bread/Corn starch/Microcrystalline cellulose and dextrose anhydrate/Lactose and magnesium stearate	SMD ^(c,d)^ = −0.17, 95% CI (−0.63, 0.29); *I*^2^ = 83.2%, *p* = 0.000

^(a)^ random effect model; studies with different probiotic formula, dose, time of intervention, etc.; ^(b)^ multi-strain probiotics; ^(c)^ single-strain probiotics; ^(d)^ in patients with Type 2 Diabetes.

**Table 3:** A table characterizing the various prebiotic compounds, along with examples of sources of these compounds from food.

Prebiotic	Example Sources	Characteristics
Fructooligosaccharides (FOS) = fructans	Onions, garlic, bananas, wheat	Compounds consisting of fructose linked to sucrose; act as reserve material in plants [50,51].
Inulin	Onions, garlic, dandelion	A group of polysaccharides belonging to the fructans, which consists of fructans linked by β-glycosidic bonds [51,62].
Resistant starch	Bread, raw potatoes, breakfast cereals	Insoluble fraction of starch that enters the large intestine unchanged, where it is fermented by intestinal bacteria [51].
Galacto-oligosaccharides (GOS)	Yogurts, modified milk, breast milk	Compounds consisting of galactose particles attached to lactose [51,52].
Lactulose	milk	Is formed by isomerization of lactose. Among other things, this process occurs when milk is heated. It is used in the treatment of constipation [60,61].
Xyloligosaccharides (XOS)	wheat bran, barley, bamboo shoots	Polymers of compounds classified as xylans, found in cereal grains [53].
β-glucans	oats, barley, mushrooms	Polysaccharides that are extracted from the cell walls of some bacteria, cereals and yeast [51,59].
Soybean oligosaccharides (SBOS)	soybean	SBOS are extracted by extracting soybeans without enzymatic treatment [54].

**Table 4:** Characteristics of the various probiotic products available for sale.

Product	Description
Sauerkraut	Can be formed naturally from chopped white cabbage with salt or with the addition of fermentation starters (*L. casei* strain). Due to the fermentation process, sauerkraut has a reduced pH, which has a preservative function. In the ready-to-eat product, the main probiotic bacteria are *Lactobacillus* and *Lactococcus*. Sauerkraut is a fermented cabbage product that may serve as a source of lactic acid bacteria with potential probiotic properties [106].
Kimchi	A type of pickled cabbage originating from traditional Korean cuisine. It consists of Chinese cabbage, onions, radishes, and spices such as ginger, garlic, salt, and large amounts of ground bell pepper. Bacteria such as *Lactobacillus plantarum* and *Lactobacillus mesenteroides* predominate in the composition. However, the health benefits of kimchi do not end with its probiotic effects in the colon. Thanks to its high content of dietary fiber, B vitamins, A, C, E, and numerous biologically active compounds, such as capsaicin, chlorophyll, and gingerol, this Korean pickle can have beneficial effects on the entire body. It has anticancer, antioxidant, and antimutagenic effects. Lowers the concentration of lipids and glucose in the blood. Influences immunity and weight maintenance [107].
Kombucha	A tea-based drink, usually black tea, that has undergone a fermentation process. Kombucha is made by adding sugar and a mixture of yeast and probiotic bacteria called SCOBY to the tea infusion. SCOBY consists of yeast from the genus *Saccharomyces* and *Zygosaccharomyces* and bacteria from the genus *Acetobacter*, *Gluconobacter*, *Lactobacillus*, and *Lactococcus*. Beverage preservatives formed during fermentation include acetic acid, lactic acid, and ethanol [108].
Tempeh	Originating from Indonesia soybean seeds previously soaked and cooked. Their fermentation occurs under the influence of the fungus *Rhizopus oligoporus*. Because of its fungal aftertaste obtained during fermentation, it is used as a vegan substitute for meat and also dairy [109].
Natto	Fermented seeds of cooked soybeans. However, its fermentation uses a special strain of *Bacillus subtilis* var. Natto, which produces nattokinase in the finished food product. Natto is a traditional fermented soybean product. In a randomized crossover study, gamma-polyglutamic acid-rich natto was shown to suppress the early postprandial blood glucose response [110].
Miso	Japanese soybeans paste that undergoes fermentation by positive starter bacteria *Aspergillus oryzae*. Miso paste is used to prepare a traditional Japanese soup. Long-term intake of miso soup has been associated with reduced nighttime blood pressure; however, evidence regarding its direct effects on gut microbiota remains limited [111].
Pickled olives	Fermented fruit of the olive tree, a delicacy of Mediterranean cuisine. A sizable group of bacteria from the *Lactobacillus* family and also fungi from the genera *Pichia*, *Candida*, and *Saccharomyces* have been isolated from them. Work is currently underway to increase the microbiological stability of this product by adding probiotic bacteria [112].

**Table 5:** Characteristics of common fermented dairy products that are used to improve the condition of gut microbiota.

Product	Description
Kefir	Originating from the Caucasus sour drink made from fermented milk. The fermentation process is induced by kefir grains consisting of lactic and acetic acid bacteria and lactose-fermenting yeast *Kluyveromyces marxianus* and non-fermenting yeast such as *Saccharomyces cerevisiae*. Kefir is characterized by a complex microbiota composed mainly of lactic acid bacteria, acetic acid bacteria, and yeasts, which contribute to its nutritional and potential health-promoting properties [113]
Yogurt	A product formed from the fermentation of raw or pasteurized milk to which the starter bacteria *Streptococcus thermophilus* and *Lactobacillus delbrueckii* have been added. The acetaldehyde and lactic acid formed during fermentation condition the slightly sour taste of yogurt. Yogurts may also be enriched with selected probiotic strains, and probiotic yogurt consumption has been investigated in relation to selected health outcomes [114].
Cheese	Some cheeses contain particular strains of bacteria that show beneficial effects on the body. These include some semi-hard and hard cheeses. However, due to their high content of saturated fatty acids and energy density, they should be eaten in limited quantities [115].

## Data Availability

No new data were created or analyzed in this study. Data sharing is not applicable to this article.
